# Development and Validation of a UPLC-MS/MS Method for the Quantitative Determination and Pharmacokinetic Analysis of Cirsimarin in Rat Plasma

**DOI:** 10.1155/2021/9953664

**Published:** 2021-06-08

**Authors:** En Zhang, Ying Wang, Fuyi Xie, Xinlei Zhuang, Xianqin Wang, Xiaomin Yu

**Affiliations:** ^1^Clinical Laboratory, Ningbo Medical Treatment Center Lihuili Hospital, Ningbo, China; ^2^Pharmacy Department, Ningbo Medical Treatment Center Lihuili Hospital, Ningbo, China; ^3^School of Pharmaceutical Sciences, Wenzhou Medical University, Wenzhou, China

## Abstract

Cirsimarin is a bioactive antilipogenic flavonoid isolated from the cotyledons of *Abrus precatorius* and represents one of the most abundant flavonoids present in this plant species. Cirsimarin exhibits excellent antioxidant, lipolysis, and other biological properties; it can effectively trigger lipid movement and demonstrates antiobesity effects. In this work, an ultra-high-performance liquid chromatography tandem mass spectrometry (UPLC-MS/MS) method was developed for the determination of cirsimarin in rat plasma after intravenous administration. A standard curve of cirsimarin in blank rat plasma was generated over the concentration range of 1–3000 ng/mL. Six rats were administered cirsimarin intravenously (1 mg/kg). The method only required 50 *μ*L of plasma for sample preparation, and the plasma proteins were precipitated with acetonitrile to pretreat the plasma sample. The precisions of cirsimarin in rat plasma were less than 14%, while the accuracies varied between 92.5% and 107.3%. In addition, the matrix effect varied between 103.6% and 107.4%, while the recoveries were greater than 84.2%. This UPLC-MS/MS method was then applied in measuring the pharmacokinetics of cirsimarin in rats. The AUC_(0-*t*)_ values of cirsimarin from the pharmacokinetic analysis were 1068.2 ± 359.2 ng/mL·h for intravenous administration. The half-life (*t*_1/2_) was 1.1 ± 0.4 h (intravenous), indicating that the metabolism of the compound was quick in the rats. Exploring the pharmacokinetics of cirsimarin *in vivo* can help better understand its metabolism.

## 1. Introduction

*Abrus precatorius* is an herbaceous flowering plant whose extract can promote body fluid, moisten the lungs, clear away heat, promote diuresis, and so on. It is used for the prevention and treatment of hepatitis, bronchitis, sore throats, and other diseases/illnesses [1, 2]. The roots, stems, and leaves of *Abrus precatorius* are often used as herbal medicines [3, 4]. Because the cotyledons of *Abrus precatorius* have a sweet taste, they are often used in the preparation of Guangdong herbal tea. Many people use them to make soup or tea as a means to help protect the liver and reduce blood fat. The cotyledons of *Abrus precatorius* contain various biologically active compounds, such as triterpenoids, alkaloids, and flavonoids [5, 6]. Cirsimarin is a bioactive antilipogenic flavonoid isolated from the cotyledons of *Abrus precatorius* and is one of the most abundant flavonoids presented in this plant species [7]. Modern pharmacological studies have shown that cirsimarin exhibits excellent antioxidant, lipolysis, and other biological properties; it can effectively trigger lipid movement and demonstrates antiobesity effects [8–10]. The performance of cirsimarin-mediated lipolysis is 20 times higher than the respective performance of caffeine. *In vivo* pharmacokinetic analysis plays an important role in determining the efficacy, mechanism of action, and clinical rationale of a drug. Therefore, it is particularly important to perform the pharmacokinetic analysis of cirsimarin *in vivo* and determine its pharmacokinetic parameters (*C*_max_, AUC, *T*_max_, etc.).

UPLC-MS/MS has the advantages of a high sensitivity, low detection limit, and minimal sample consumption, and it is widely employed in the analysis of chemical components, elucidation of drug metabolism, impurity identification, and other drug analyses [11–14]. To the best of our knowledge, no study has reported the quantitation of cirsimarin in plasma by UPLC-MS/MS. Therefore, we established herein a UPLC-MS/MS method for the determination of cirsimarin in rat plasma and applied this method to measure the plasma concentration and pharmacokinetics of cirsimarin.

## 2. Materials and Methods

### 2.1. Chemical

**Figure 1:**
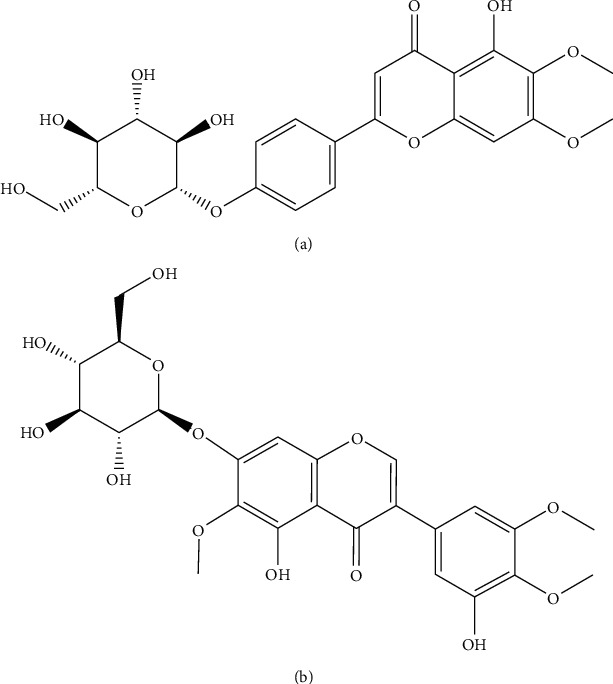
Chemical structures of cirsimarin (a) and iridin (IS) (b).

Cirsimarin (batch number: MUST-20041802, purity > 98%, Figure 1(a)) and iridin (batch number: MUST-19070204, internal standard (IS), purity > 98%, Figure 1(b)) were obtained from Chengdu Mansite Biotechnology Co., Ltd (Chengdu, China). Water was purified by the Millipore Milli-Q system (Bedford, MA, USA). HPLC-grade acetonitrile, methanol, and formic acid were obtained from Tedia Company (Ohio, USA).

### 2.2. Instrumentation and Methods

**Figure 2:**
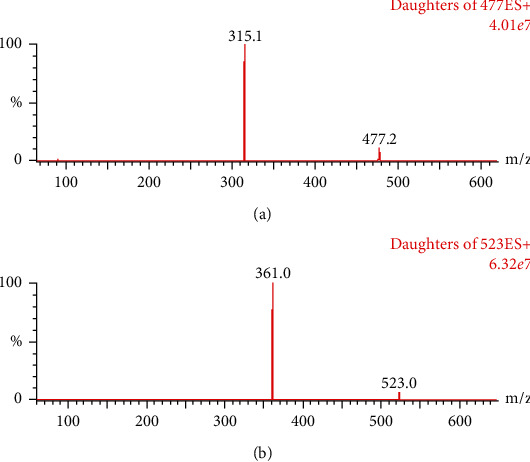
Mass spectra of cirsimarin (a) and IS (b).

An ACQUITY H-Class UPLC equipped with a XEVO TQS-micro triple quadrupole mass spectrometer (Waters Corp., Milford, MA, USA) was utilized in this work. A UPLC BEH C18 column (2.1 mm × 50 mm, 1.7 *μ*m) was used for the separation of the analytes and was maintained at 40°C. The chromatographic method entailed a gradient elution of the mobile phase, which consisted of acetonitrile and water with 0.1% formic acid (0.4 mL/min), with the following profile: 0–0.2 min, 10% acetonitrile isocratic; 0.2–1.2 min, 10–90% acetonitrile; 1.2–2.0 min, 90% acetonitrile isocratic; 2.0–2.2 min, 90–10% acetonitrile; and 2.2–3.0 min, 10% acetonitrile isocratic. The capillary voltage was set to 2.2 kV, the ion source temperature was 150°C, and the desolvation temperature was 450°C. Nitrogen gas was used for desolvation (900 L/h) and nebulization. The analytes were detected in the multiple reaction monitoring (MRM) mode and ionized in ESI-positive mode by monitoring the transitions of *m*/*z*477⟶315 for cirsimarin (cone voltage: 14 V; collision energy: 18 V) and *m*/*z*523⟶361 for the IS (cone voltage: 10 V; collision energy: 14 V) (Figure 2).

### 2.3. Stock and Working Solutions

Stock solutions of cirsimarin (1.0 mg/mL) and iridin (1.0 mg/mL) were prepared in methanol. Working solutions of cirsimarin were then prepared at different concentrations (10, 50, 200, 400, 1000, 2000, 4000, 8000, 15,000, and 30,000 ng/mL) by diluting the stock solution with methanol.

### 2.4. Standard Curve

Blank rat plasma was spiked with the working solutions of cirsimarin to prepare standard solutions with concentrations of 1, 5, 20, 40, 100, 200, 400, 800, 1500, and 3000 ng/mL of cirsimarin. Quality control samples (4, 180, and 2500 ng/mL) were also prepared using the same method.

### 2.5. Plasma Preparation

In a 1.5 mL centrifuge tube, 50 *μ*L of rat plasma was diluted into 150 *μ*L of acetonitrile (containing 50 ng/mL of IS), and the tube was vortexed for 1.0 min and centrifuged at 13,000 rpm for 10 min at 4°C. The resulting supernatant (100 *μ*L) was transferred to an LC-MS vial, and 2 *μ*L of the solution was injected into the UPLC-MS/MS for analysis.

### 2.6. Pharmacokinetics

Sprague Dawley (SD) rats (male, 200–220 g in weight) were obtained from the Animal Experimental Center of Wenzhou Medical University (Wenzhou, China). All experimental procedures were approved by the Animal Care Committee of Wenzhou Medical University (Wydw 2019-0982). Blood (150 *μ*L) was collected from the tail vein at 0.083, 0.5, 1, 2, 3, 4, 6, and 8 h after the intravenous administration of cirsimarin (1 mg/kg) and transferred to a centrifuge tube containing heparin. After centrifuging at 3000 rpm for 10 min, the plasma was removed and stored at –20°C. The pharmacokinetic parameters were then analyzed using the DAS 2.0 software package (China Pharmaceutical University).

## 3. Results

### 3.1. Method Validation

**Figure 3:**
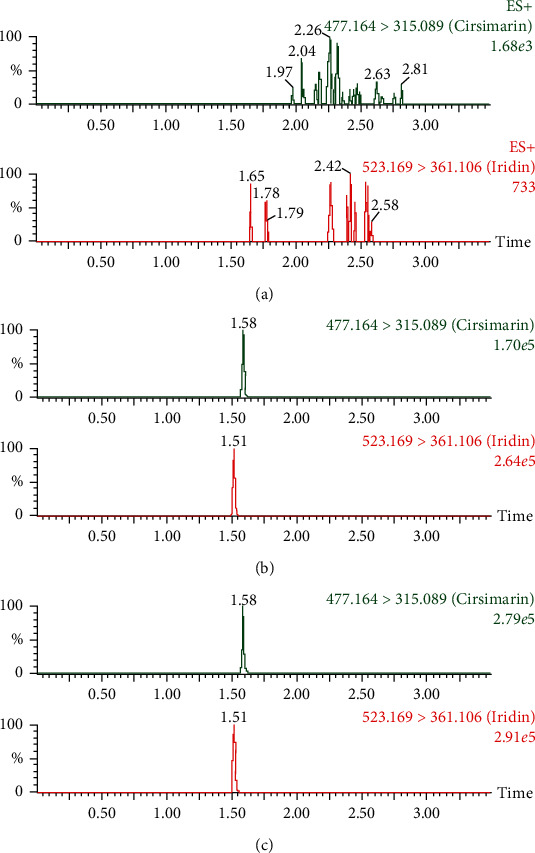
UPLC-MS/MS chromatograms of cirsimarin and IS in rat plasma: (a) blank rat plasma, (b) blank rat plasma spiked with cirsimarin and IS, and (c) rat plasma after intravenous administration of cirsimarin.

The selectivity of the method was evaluated by analyzing blank rat plasma, blank rat plasma spiked with cirsimarin and IS, and extracted rat plasma samples after administration of cirsimarin. As shown in Figure 3, there were no obvious impurities or endogenous substances that had intervened in the detection of cirsimarin and IS.

A calibration curve was generated over the concentration range of 1–3000 ng/mL with a weighting factor of the reciprocal of the concentration (1/*x*). The lower limit of quantification (LLOQ) was defined as the lowest concentration measured in the calibration curves. The resulting standard curve equation was *y* = 0.0046*x* − 0.0034 (*r* = 0.9994), where *y* was the ratio of peak area of cirsimarin to internal standard and *x* represented the cirsimarin concentrations in rat plasma. The LLOQ was determined to be 1 ng/mL. Limit of detection (LOD) was determined to be 0.3 ng/mL, with a signal-to-noise ratio of 3.

The accuracy and precision of the method were assessed by the measurement of three QC samples (4, 180, and 2500 ng/mL) in six replicates over three days. The recovery of cirsimarin was evaluated by comparing the peak areas of the extracted QC samples to the peak areas of the reference QC solutions of the same concentration prepared in blank rat plasma (*n* = 6). To evaluate the matrix effect of the rat plasma, blank plasma was extracted from the rat and supplemented with the cirsimarin analyte at concentrations of 4, 180, and 2500 ng/mL. The corresponding peak areas in the chromatograms were then compared to the peak areas of the neat standard solutions at equivalent concentrations. The intraday and interday precisions of cirsimarin in rat plasma were all less than 14%. The corresponding accuracies were between 92.5% and 107.3%, the matrix effect values were between 103.6% and 107.4%, and the recoveries were all greater than 84.2% (Table 1).

Carry-over was assessed following the injection of blank rat plasma immediately after three repeat injections of rat plasma containing cirsimarin for the determination of the upper limit of quantification (ULOQ) of the method [15]. No significant peak related to cirsimarin was identified in the chromatogram (≥20% of the LLOQ and 5% of the IS) of the blank samples injected after the ULOQ samples. Adding an extra 0.5 min to the end of the chromatography method effectively flushed the column and system, thereby eliminating any carry-over [15].

Stability values of cirsimarin in rat plasma were evaluated by analyzing three replicates of rat plasma samples (4, 180, and 2500 ng/mL) that were all exposed to different conditions. One sample was stored at room temperature for 2 h; another sample underwent three freeze-thaw cycles, was processed, and then stored at room temperature for 12 h; and the third sample was stored at –20°C for 30 days. After analysis of these plasma samples, it was found that the variation between the samples was between 92.3% and 106.8%, and the corresponding RSD was less than 14% (Table 2).

### 3.2. Pharmacokinetics

**Figure 4:**
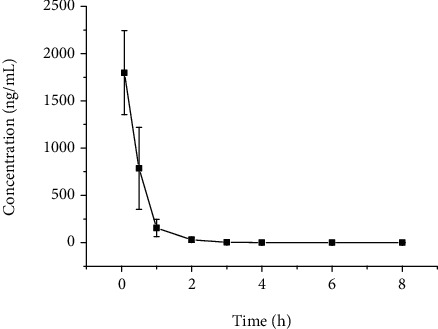
Plasma concentration-time curve of cirsimarin after intravenous administration (1 mg/kg).

The plot of the plasma concentration over time of cirsimarin is displayed in Figure 4. The main pharmacokinetic parameters analyzed by the noncompartment model are provided in Table 3. The AUC_(0-*t*)_ corresponding to the extracted rat plasma after intravenous administration of cirsimarin was 1068.2 ± 359.2 ng/mL·h.

## 4. Discussion

Extensive literature reports detail the use of HPLC, GC, LC-MS, and LC-MS/MS in the determination of drug plasma concentrations [12]. Among them, the sample analysis time by HPLC is relatively long, and the LLOQ is relatively high, both of which are not ideal for achieving the measurement requirements of cirsimarin. The use of GC requires high temperatures, which can easily degrade cirsimarin, thus affecting the accuracy of the measurement. The LLOQ of LC-MS/MS is lower than HPLC, but the sample analysis time can be as long as 10 min, which is not conducive to a rapid, efficient, and sensitive sample analysis. However, the UPLC-MS/MS method established in this work successfully overcame the above shortcomings to achieve a rapid, efficient, and sensitive analysis of the rat plasma samples.

In this work, the mass spectrometry and liquid chromatography conditions were optimized. Cirsimarin is a weakly basic compound, which makes it suitable for running the mass spectrometry analysis in positive-ion mode. Adding formic acid to the mobile phase enabled protonation. During the optimization of the method, it was found that the *m*/*z* 315 fragment abundance was higher when the cone voltage was greater than 12 V and when the collision energy was less than 18 V. Therefore, a cone voltage of 14 V and a collision energy of 18 V were utilized for quantitative analysis in order to achieve the highest sensitivity for the *m*/*z*477 ⟶ 315 transition of cirsimarin. When isocratic acetonitrile-water (1 : 1 *v*/*v*) and acetonitrile-0.1% formic acid in water (1 : 1 *v*/*v*) were used as the mobile phase, broad peaks appeared in the chromatograms. Considering the interferences from biological impurities in the rat plasma samples, a gradient elution method using acetonitrile-0.1% formic acid in water was selected as the optimal mobile phase for the liquid chromatographic analysis. Meanwhile, the method was optimized to shorten the retention time of cirsimarin and the IS to less than 3 min in order to make the method more rapid. By optimizing the *m*/*z*477 ⟶ 315 transition for cirsimarin and the mobile phase elution gradient conditions, the sensitivity and stability were significantly improved compared to the conditions of the initial methods.

The method for the pretreatment of a plasma sample has a decisive influence on the accuracy of the drug concentration determination in the plasma [16, 17]. Therefore, the treatment of a plasma sample is particularly important. In the early stages of this experiment, the effects of methanol, acetonitrile, and a mixture of methanol : acetonitrile (1 : 1 *v*/*v*) on the ability to directly precipitate plasma proteins were assessed. The results showed that there was no significant difference in the precipitation of proteins between methanol, acetonitrile, and the 1 : 1 *v*/*v* mixture of these two solvents; however, when the proportion of organic phase to plasma was increased (e.g., plasma : organic phase  = 1 : 3), the extraction recovery increased to between 80% and 100%. Moreover, if the proteins were precipitated using a lower proportion of organic phase to plasma (e.g., 1 : 1 or 1 : 2), the proteins in the plasma would not precipitate completely, which would cause the unprecipitated proteins to easily clog the chromatography column. When ethyl acetate was used to extract the plasma, the recovery was less than 60%. This might have been due to the fact that ethyl acetate could not denature the proteins, and many drugs remained in the plasma. We tried a different solvent for pretreatment, and protein precipitation by acetonitrile (1/3, *v*/*v*) was reasonable and feasible.

For the selection of the IS, we chose iridin as the IS because it has a similar structure to cirsimarin [18, 19]. During the plasma analysis, it was found that the retention time of iridin was shorter, there was no crossinterference between iridin and cirsimarin, and the extraction recovery of iridin was as high as 90%. In addition, the matrix interference was almost negligible, and the mass response was very stable, which is why iridin was selected as the internal standard.

UPLC-MS/MS was utilized in the quantitative analysis of cirsimarin in rat plasma because it enabled much faster analyses than traditional HPLC. Cirsimarin was eliminated from the plasma with a *t*_1/2_ of 1.1 ± 0.4 h after intravenous administration. These results indicated that plasma metabolism was fast in rats. The AUC_(0-*t*)_ values were 1068.2 ± 359.2 ng/mL·h for intravenous administration. The pharmacokinetic analysis of cirsimarin *in vivo* could help better understand its metabolism.

## 5. Conclusion

In this study, we developed a simple, rapid, and selective UPLC-MS/MS method for the measurement of cirsimarin in rat plasma, of which the LLOQ was 1 ng/mL. The analysis time for one sample only required 3 min. Pretreatment of the plasma sample before injection was achieved with acetonitrile, and the method only required 50 *μ*L of treated plasma for accurate analysis. This method was then applied in the pharmacokinetic analysis of cirsimarin in rats, and it represents the first reported method for the measurement of cirsimarin concentrations *in vivo*.

## Figures and Tables

**Table 1:** Accuracy, precision, matrix effect, and recovery of cirsimarin in rat plasma (n =6).

Concentration (ng/mL)	Accuracy (%)		Precision (RSD %)		Matrix effect (%)	Recovery (%)
	Intraday	Interday	Intraday	Interday		
1	92.5	107.3	10.5	13.6	104.7	92.8
4	101.8	99.4	3.4	6.9	106.6	84.2
180	105.2	103.9	4.5	7.3	107.4	95.3
2500	101.0	97.1	8.4	2.7	103.6	98.0

**Table 2:** Summary of the stability of cirsimarin under various storage conditions (*n* = 3).

Concentration (ng/mL)	Autosampler (4°C, 12 h)		Ambient (2 h)		–20°C (30 d)		Freeze-thaw	
	Accuracy	RSD	Accuracy	RSD	Accuracy	RSD	Accuracy	RSD
4	106.8	5.1	101.8	5.6	106.8	7.8	104.4	13.8
180	99.0	4.2	97.1	9.9	99.0	11.1	92.3	5.6
2500	106.4	6.8	101.5	5.4	106.4	5.7	92.9	3.4

**Table 3:** Main pharmacokinetic parameters of cirsimarin in rats.

Parameters	Unit	IV (1 mg/kg)
AUC_(0-*t*)_	ng/mL·h	1068.0 ± 359.2
AUC_(0-∞)_	ng/mL·h	1068.2 ± 359.2
MRT_(0-*t*)_	h	0.4 ± 0.1
MRT_(0-∞)_	h	0.4 ± 0.1
*t*_1/2*z*_	h	1.1 ± 0.4
CLz	L/h/kg	1.0 ± 0.3
Vz	L/kg	1.8 ± 1.2
*C*_max_	ng/mL	1798.5 ± 444.1

## Data Availability

The data used to support the findings of this study are included within the article.
